# An update on Burkitt lymphoma: a review of pathogenesis and multimodality imaging assessment of disease presentation, treatment response, and recurrence

**DOI:** 10.1186/s13244-019-0733-7

**Published:** 2019-05-21

**Authors:** Kevin Kalisz, Francesco Alessandrino, Rose Beck, Daniel Smith, Elias Kikano, Nikhil H. Ramaiya, Sree Harsha Tirumani

**Affiliations:** 1 Department of Radiology, Duke University, Durham, NC USA; 2 Department of Imaging, Dana Farber Cancer Institute, Harvard Medical School, 450 Brookline Avenue, Boston, MA 02215 USA; 3 Department of Radiology, Brigham and Women’s Hospital, Harvard Medical School, Boston, MA USA; 4 Department of Pathology, UH Cleveland Medical Center, Case Western Reserve University, Cleveland, OH USA; 5 Department of Radiology, UH Cleveland Medical Center, Case Western Reserve University, Cleveland, OH USA

**Keywords:** Burkitt lymphoma, Lymphoma, B cell, Diagnostic imaging, Computed tomography, Drug therapy

## Abstract

Burkitt lymphoma (BL) is a highly aggressive, rapidly growing B cell non-Hodgkin lymphoma, which manifests in several subtypes including sporadic, endemic, and immunodeficiency-associated forms. Pathologically, BL is classically characterized by translocations of chromosomes 8 and 14 resulting in upregulation of the c-myc protein transcription factor with upregulation of cell proliferation. BL affects nearly every organ system, most commonly the abdomen and pelvis in the sporadic form. Imaging using a multimodality approach plays a crucial role in the management of BL from diagnosis, staging, and evaluation of treatment response to therapy-related complications with ultrasound, computed tomography, magnetic resonance imaging, and positron emission tomography playing roles. In this article, we review the pathobiology and classification of BL, illustrate a multimodality imaging approach in evaluating common and uncommon sites of involvement within the trunk and head and neck, and review common therapies and treatment-related complications.

## Key points

- Burkitt lymphoma can be differentiated from other forms of diffuse large B cell lymphoma based on underlying pathobiology, which is reflected in the updated WHO classification.
- Radiologists should recognize common and uncommon presentations and sites of disease to appropriately guide clinicians given the urgency of potential of treatment.
- Multiple imaging modalities play a key role in Burkitt lymphoma evaluation throughout the entire disease course, each with advantages and disadvantages.

## Introduction

Burkitt lymphoma (BL) is a highly aggressive B cell non-Hodgkin lymphoma (NHL) characterized by the translocation and deregulation of the *MYC* gene on chromosome 8 with the potential to involve multiple organ systems. Three subtypes of BL (sporadic, endemic, and immunodeficiency-associated) are recognized with different epidemiology, risk factors, and clinical presentations.

The sporadic subtype of BL is generally observed in the USA and Western Europe with an overall incidence of three cases per million persons per year in the general population. Sporadic BL is relatively more common in the pediatric population, accounting for 30% of pediatric lymphomas with a peak incidence around the age of 10 years old, while only representing less than 1% of NHL in adults [1, 2]. Sporadic cases are associated with Epstein-Barr virus (EBV), and the most common site of involvement is within the abdomen, particularly the bowel [3]. The sporadic subtype will be the focus of this article.

The endemic subtype is found in equatorial Africa and New Guinea with a near 50-fold higher incidence than that seen in the USA [4]. Endemic BL accounts for up to 50% of all childhood cancer in equatorial Africa with an estimated incidence of 3 to 6 cases per 100,000 children per year [5]. The most common presentation is a facial tumor, and nearly all cases are associated with EBV [6]. Immunodeficiency-associated BL is mostly commonly seen in HIV-positive patients, but also may be seen in allograft recipients and patients with congenital immunodeficiency. HIV patients with BL tend to have relatively higher CD4 counts (> 200 cells/μL) and the majority demonstrate EBV positivity. The most common sites of involvement are lymph nodes, bone marrow, and the central nervous system (CNS) [3].

BL exhibits very heterogeneous presentations and clinical courses among patients. In this article, we review the pathogenesis and classification of BL, illustrate a multimodality imaging approach in evaluating common and uncommon sites of involvement, and highlight important treatment topics.

## Pathology and classification

### Pathogenesis and histologic features

BL is derived from germinal center B cells. The three clinical subtypes of BL are thought to arise from B cells at different stages of their development. BL development depends on expression of *MYC* gene, which encodes for the c-myc protein transcription factor, which is located on chromosome 8q24 and regulates cell proliferation, differentiation, and apoptosis. BL is characterized by inappropriately high levels of c-myc, which can result via several different mechanisms, most commonly by translocation of the long arm of chromosome 8 (containing the *MYC* gene) and the Ig heavy chain gene on chromosome 14. c-Myc overexpression leads to rapid B cell proliferation accounting for the rapid doubling time of BL tumor cells (between 24 and 48 h) [7]. Histologically, BL demonstrates a “starry sky” appearance with benign histiocytes containing abundant, clear cytoplasm dispersed among a background of homogeneous, basophilic tumor cells. Markedly high rates of both proliferation and apoptotic cell death are generally observed [8].

Related but distinct pathologic entities are lymphomas demonstrating “double” or “triple-hit” phenomena accounting for 3–10% of diffuse large B cell lymphomas (DLBCL). “Double hit” lymphomas are characterized by the presence of both *MYC* and *BCL*-*2* (or less commonly *BCL*-*6*) rearrangements, while “triple-hit” lymphomas demonstrate *MYC*, *BCL*-*2*, and *BCL*-*6* rearrangements. These lymphoma subtypes exhibit further inhibition of apoptosis and cell survival and are associated with poorer prognosis. Multiple hit lymphomas should be distinguished from “double expressor” DLBCLs in which *MYC* and *BCL*-*2* genes are overexpressed at the protein level without the genetic rearrangements [9].

### WHO classification

In the 2016 World Health Organization (WHO) classification of lymphoid neoplasms, aggressive B cell lymphomas, which includes BL, have undergone revision now accounting for both tumor morphology and genetics [10]. Initial immunohistochemical or gene expression studies are performed to confirm a germinal center B cell-based cell origin. Further immunophenotype testing specific for BL and identification of *MYC* overexpression supports the identification of BL. However, it has been shown that some aggressive lymphoma subtypes demonstrate features overlapping BL and other DLBCLs, which had been previously classified as B cell lymphoma, unclassifiable. In the 2016 WHO classification, this category has been eliminated and replaced by the category “high-grade B cell lymphoma, with *MYC* and *BCL*-*2* and/or *BCL-6* rearrangements”, which encompasses “double” and “triple-hit” lymphomas. Additionally, entities previously falling into a “Burkitt-like” lymphoma category (i.e., aggressive B cell lymphoma entities resembling BL) are classified into either high-grade B cell lymphoma with *MYC* and *BCL*-*2* and/or *BCL*-*6* rearrangement, Burkitt-like lymphoma with 11q aberration, or high-grade B cell lymphoma, not otherwise specified. Notably, the double-expresser is not considered in these categories but is recognized as a poor prognostic sign within DLBCL.

## Imaging evaluation

Although the diagnosis of BL is confirmed pathologically by characteristic biology, immunophenotype, and genetic analysis, imaging plays a critical role throughout the entire clinical course of these patients from initial detection, including emergent presentations, to evaluating treatment response and potential complications. Various modalities are employed in the assessment of BL, each with particular strengths and weaknesses.

For patients presenting in an emergent setting with acute symptoms, either ultrasound or computed tomography (CT) is often utilized. Ultrasound is particularly useful in the pediatric population given the lack of ionizing radiation. Ultrasound can evaluate for the presence of palpable masses or intussusception in pediatric patients and is well suited to evaluate small and superficial sites of involvement such as peripheral lymph nodes, gonads, thyroid, and breast [11]. Ultrasound is appropriate for the evaluation of lymph node architecture and assessing benign versus malignant nodal features. Ultrasound findings or malignant nodes include loss of an echogenic hilum, asymmetric cortical thickening, distortion of intranodal vascular architecture (i.e., aberrant vessels), and amputated short subcapsular vessels. However, ultrasound may be limited by operator dependence and in its evaluation of deeper abdominal structures, particularly in larger adults. Conversely, CT offers rapid, whole body evaluation (including staging information) with improved soft tissue resolution while overcoming the acoustic limitations of ultrasound [12]. Exams are preferably performed with intravenous contrast for better soft tissue evaluation, although poor renal function and allergy may prohibit its use. Multiplanar reformatted images should routinely be used. In the setting of osseous involvement, CT is adequate to evaluate for bone destruction. A principle disadvantage of CT is ionizing radiation exposure. It should be emphasized that modalities utilizing ionizing radiation should be performed in accordance with the principle of As Low As Reasonably Achievable (ALARA). Furthermore, interpretation of CT in some pediatric patients may be difficult in the setting of absent intra-abdominal fat planes.

Although not typically employed in the emergency setting, magnetic resonance imaging (MRI) has an increasing role in the evaluation of BL. The strength of MRI is its superior soft tissue characterization, which is of particular value in assessing tumor extension and central nervous system (CNS) involvement. Standard protocols utilize a combination of multiplanar T1- and T2-weighted images, with and without fat saturation, although protocols should be tailored to the anatomy being evaluated. For example, if there is clinical concern for biliary obstruction, magnetic resonance cholangiopancreatography (MRCP) can be obtained. Although data specific to Burkitt lymphoma is lacking, the addition of diffusion-weighted imaging (DWI) has demonstrated added value over conventional sequences by increasing lesion conspicuity and improving accuracy of initial diagnosis in other lymphoma subtypes [13]. Furthermore, DWI in lymphoma has shown to correlate with higher cellularity and proliferative index and has shown utility in assessing treatment response [14, 15]. The lack of ionizing radiation is an advantage in assessing pediatric patients, particularly those requiring serial follow-up imaging. However, limitations of MRI include long exam times and relatively limited availability, particularly in emergent settings.

Positron emission tomography (PET), when combined with CT, offers the advantage of combining functional information with anatomic imaging. There is a growing body of literature evaluating the utility of PET/CT in both pediatric and adult BL patients (Table 1) [16–22]. For example, Carrillo-Cruz et al. demonstrated a high discrepancy rate between CT and PET/CT with PET/CT achieving a 100% negative predictive value in predicting treatment response as well 100% positive predictive values in predicting recurrence using a standardized uptake value (SUV) change threshold less than 66% [19]. Similarly, Wei et al. showed significant reduction in SUV_max_ on interim and post-therapy PET/CT and that changes in SUV_max_ greater than 50% was a favorable cutoff point to predict the overall survival of BL patients [22]. PET/CT may also offer superior staging compared to anatomic imaging such as CT. For example, in the pediatric population, PET/CT has demonstrated better ability to detect both nodal and extranodal sites of disease and greater impact on initial staging compared to CT [23]. PET/MRI has not yet been investigated in BL patients, although has shown similar performance as PET/CT in staging and follow-up in other lymphoma cohorts [24]. PET/MRI can simultaneously combine soft tissue characterization strengths of MRI with the functional assessment of PET. Furthermore, substitution of the CT component with MRI allows for radiation dose savings. However, both PET/CT and PET/MRI are subject to relatively long exams and limited availability, particularly PET/MRI. Also, PET exams are subject to added radiation exposure.

**Table 1:** Utility of PET in Burkitt lymphoma

Study (year published)	Patient population (cohort size)	Utility
Albano et al. (2018) [16]	Adults (*n* = 65)	-End of treatment PET/CT results significantly correlate with PFS and OS -Interim PET/CT did not correlate with PFS and OS
Albano et al. (2019) [17]	Adults (*n* = 65)	-Total metabolic tumor volume and total lesion glycolysis independent prognostic factors for PFS and OS
Bailly et al. (2014) [18]	Children (*n* = 19)	-Significantly higher NPV of PET (93%) compared to conventional imaging (73%) in detecting CR -PFS significantly higher in patients with negative PET than those with positive PET
Carrillo-Cruz et al. (2015) [19]	Adults and children (*n* = 32)	−100% NPV in predicting CR −100% PPV of nonresponse with SUV change < 66% after treatment
Davidson et al. (2018) [20]	Adults (*n* = 20)	-Increased splenic FDG uptake rarely involved at time of staging -Low rate of spleen involvement may serve as a specific characteristic of BL
Karantanis et al. (2010) [21]	Adults and children (*n* = 15)	-High sensitivity (100%) and specificity (94–96%) for detection of nodal and extranodal disease
Wei et al. (2015) [22]	Adults (*n* = 29)	-Significant reduction in SUV_max_ during interim and post-therapy PET/CTs -SUV decease > 50% after post-therapy PET/CT was a favorable cutoff point to predict OS

*PFS* progression-free survival, *OS* overall survival, *NPV* negative predictive value, *PPV* positive predictive value, *CR* complete response

According to the National Comprehensive Cancer Network (NCCN) guidelines, initial staging in adults should be performed with diagnostic CT of the chest, abdomen, and pelvis [25]. Initial imaging studies in children with BL must include chest radiograph, cervical and abdominal ultrasound, CT of the chest, abdomen, and pelvis. Depending on the clinical presentation, contrast-enhanced neck and/or brain MRI may also be warranted. PET/CT should be performed if it is possible, provided it will not delay treatment, which should be started promptly, given the rapid tumor growth [26]. Also, initial cardiac function evaluation should be performed with either echocardiogram or multi-gated (MUGA) cardiac blood pool scan in anticipation of treatment with an anthracycline or anthracenedione-based regimen. Initial staging PET/CT may be useful, although is not currently routinely recommended, although it should not delay prompt treatment start. Depending on the clinical presentation, contrast-enhanced neck and/or brain MRI may also be warranted. The role of PET/CT imaging prior to completion of therapy has not been well-established. Contrary to evidence demonstrating prognostic value of interim PET/CT in DLBCL, studies to date evaluating this practice in BL have not shown similar results [16, 22, 27]. If there is a concern for relapsed or refractory disease, PET/CT is recommended as the modality of choice.

## Imaging findings

### Abdomen and pelvis

**Fig. 1:**
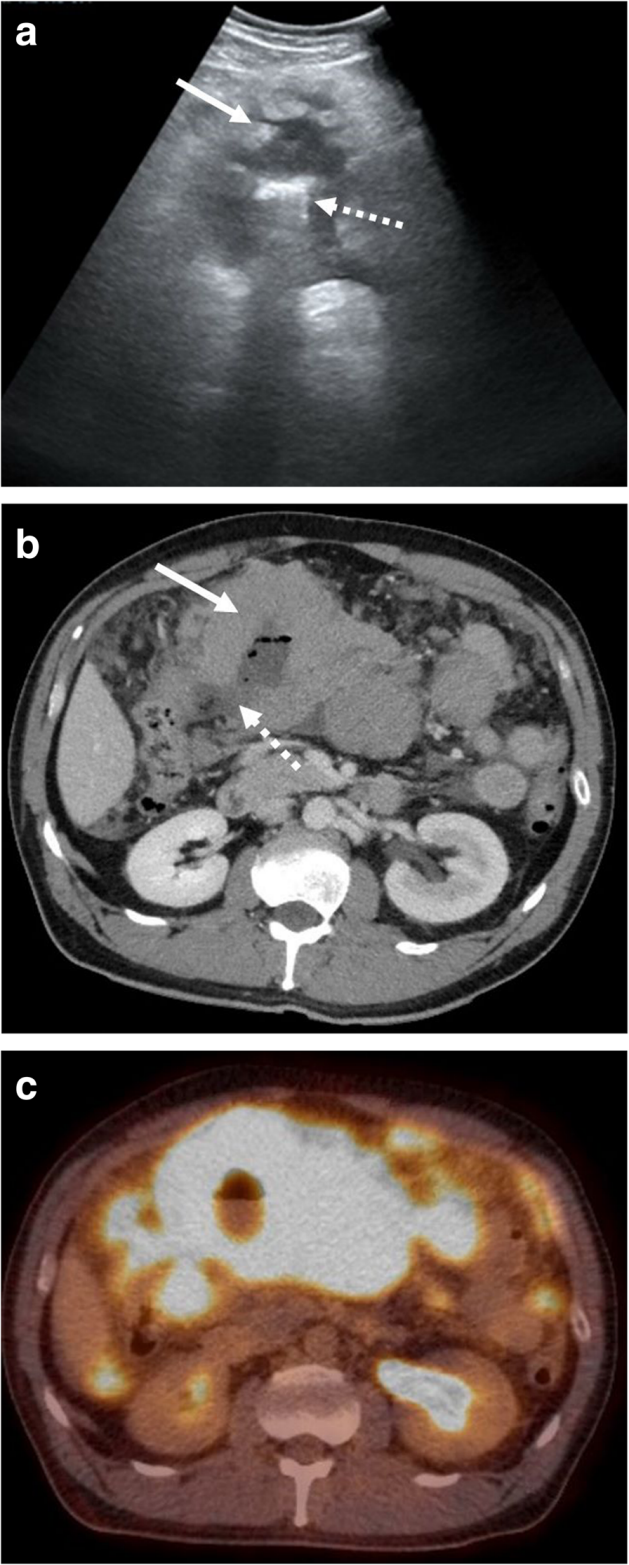
Bowel involvement with perforation. A 49-year-old male presenting to the emergency department with abdominal pain. **a** Transverse grayscale ultrasound image demonstrates mass-like thickening of a bowel loop within the mid-abdomen (solid arrow) with central air and associated dirty shadowing (dashed arrow). **b** Subsequently performed axial CT shows a large central mass (solid arrow) with associated perforation (dashed arrow). **c** Follow-up fused axial PET-CT demonstrates associated hypermetabolic activity and extensive background abdominal disease. Burkitt lymphoma was confirmed at surgery

**Fig. 2:**
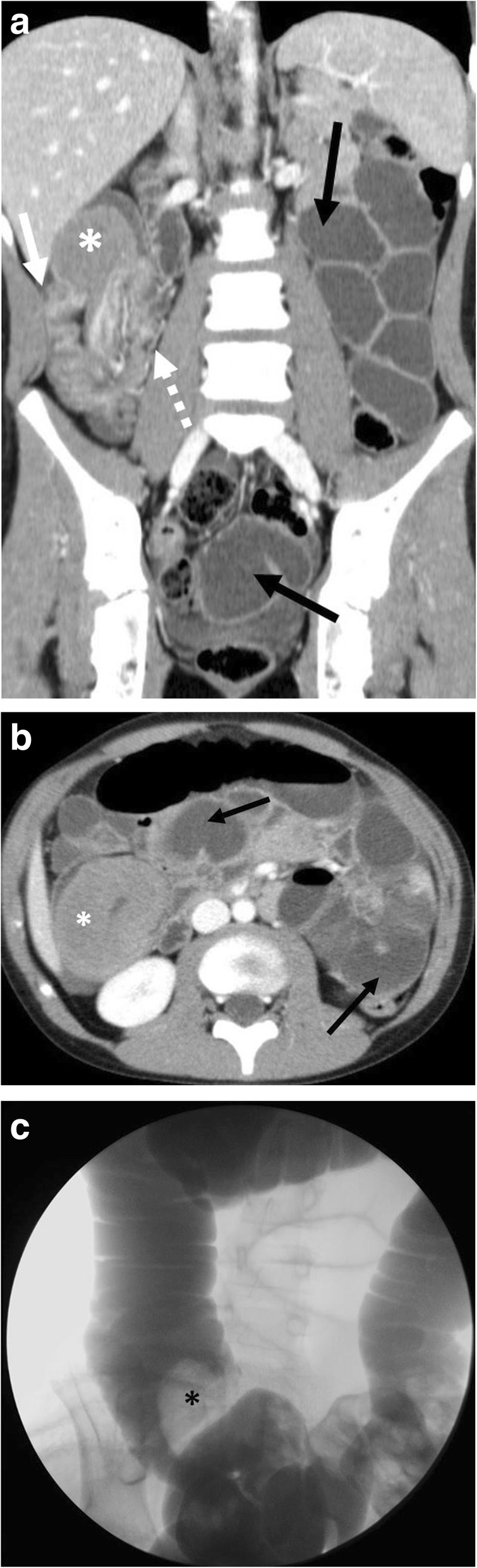
Bowel involvement with intussusception. A 10-year-old male presenting to the emergency department with abdominal pain. **a** Coronal and (**b**) axial contrast enhanced CT images demonstrate ileo-colic intussusception with intussusceptum (dashed arrow) and intussuscipiens (solid arrow) and dilated, fluid-filled small bowel loops (black arrows) from an associated small bowel obstruction. Lead point mass (asterisks) is also seen. **c** Subsequent fluoroscopic-guided contrast reduction enema demonstrating filling defect (asterisks) corresponding to the lead point mass was ultimately unsuccessful. The patient underwent surgical resection with pathology demonstrating Burkitt lymphoma

**Fig. 3:**
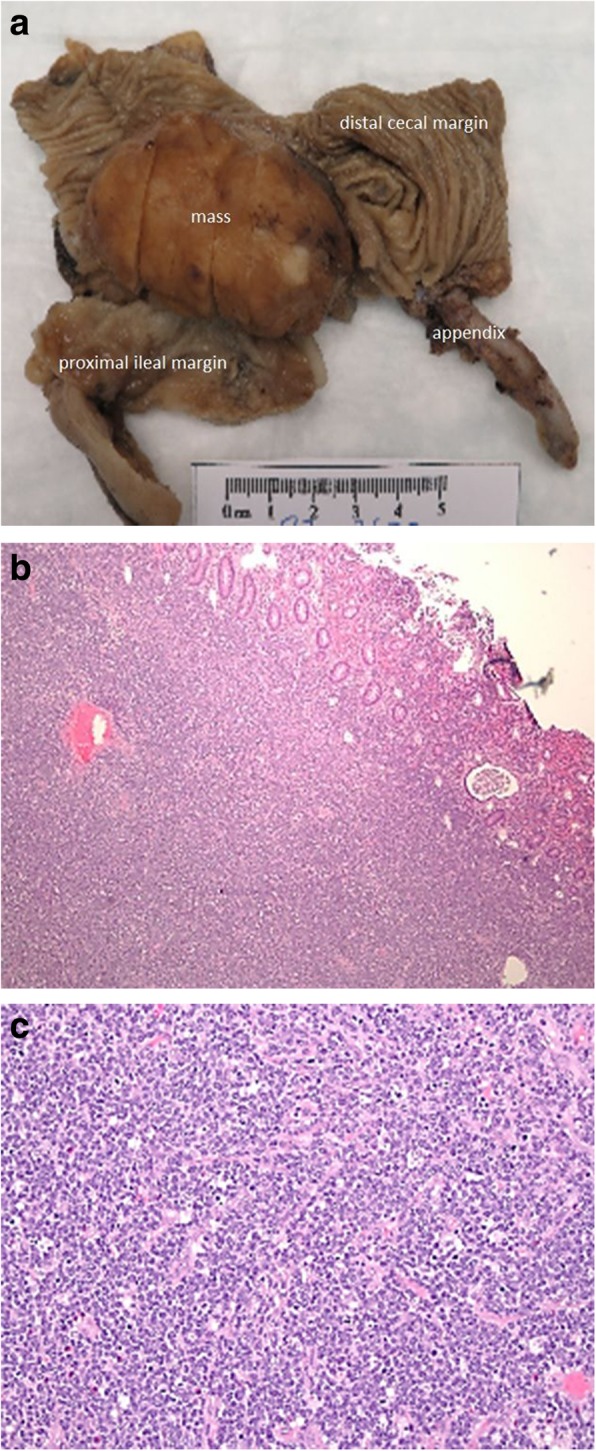
Pathology findings of Burkitt lymphoma. A 10-year-old male (presented in Fig. 2). **a** Gross exam shows a 6 cm firm mass at the ileo-cecal junction. Microscopically, (**b**) (× 40) the lymphoma is composed of sheets of monotonous lymphocytes invading the bowel mucosa and cells (**c**) (× 200) has a characteristic “starry sky” appearance due to the presence of scattered histiocytes engulfing apoptotic lymphoma

The abdomen is the most common site of involvement of BL. The most frequently involved organ is the small bowel at the ileo-cecal region due to the high concentration of lymphoid tissue. BL of the stomach and appendix are uncommon [28]. BL of the bowel appears as either a focal mass or segmental wall thickening. Wall thickening has been described as aneurysmal, with associated dilation of the lumen. Obstruction, different from other bowel malignancies and masses, is uncommon. Masses communicating with the bowel lumen may demonstrate internal air, which is best appreciated on CT but also may be identified on ultrasound by the presence of “dirty shadowing” (Fig. 1). Intussusception leading to bowel obstruction is a well-documented complication of bowel involvement and reason for emergent presentation, particularly in the pediatric population (Figs. 2 and 3) [29]. On ultrasound, signs of intussusception include concentric alternating echogenic and hypoechoic bands of bowel layers (“target sign”) on transverse views and corresponding “pseudokidney” sign on longitudinal views. CT demonstrates telescoping bowel with associated mesenteric fat and vessels and better depicts the extent of an upstream bowel obstruction. The BL mass lead point may or may not be discretely visualized on initial imaging.

**Fig. 4:**
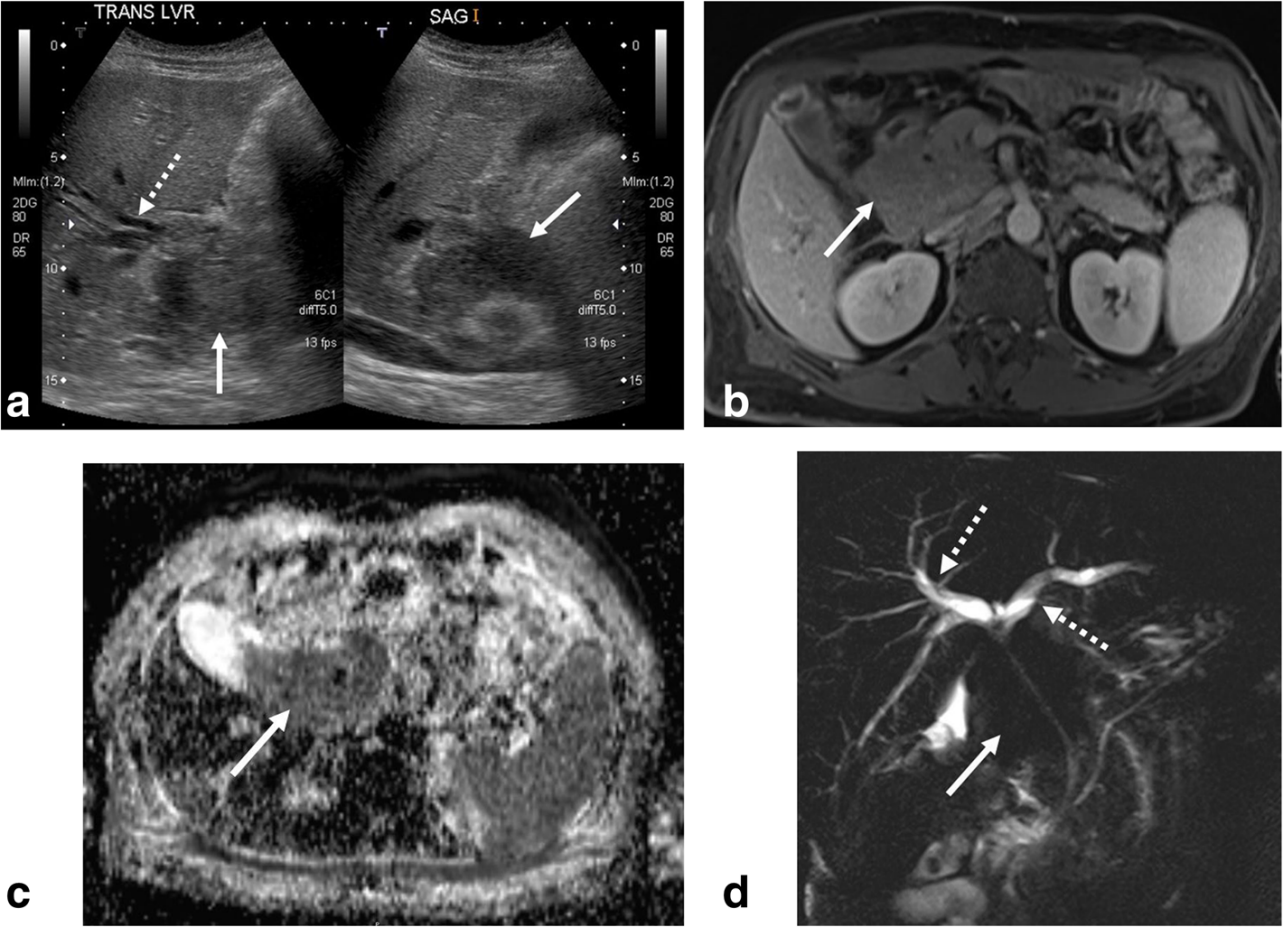
Nodal mass with biliary obstruction. A 55-year-old male presenting with jaundice. **a** Initial grayscale transverse and sagittal ultrasound images demonstrate an ill-defined hypoechoic mass near the porta hepatis (solid arrow) with associated biliary dilatation. Follow-up MRI with (**b**) axial post contrast, (**c**) apparent diffusion coefficient map, and (**d**) coronal MRCP images demonstrate an enhancing mass with diffusion restriction (solid arrows) encasing and narrowing the extrahepatic bile ducts causing intrahepatic biliary dilatation (dashed arrow)

BL also presents within the abdomen as mesenteric and retroperitoneal nodes. These may be single or multiple and may grow to conglomerate masses that often encase and grow along major mesenteric vessels with the potential to cause biliary, urinary, or bowel obstruction (Fig. 4) [12]. Ascites is also a potential finding in patients with abdominal involvement [28]. In advanced cases, lymphomatosis may be present appearing as multiple peritoneal nodules. However, isolated involvement of the peritoneum is rare [30].

**Fig. 5:**
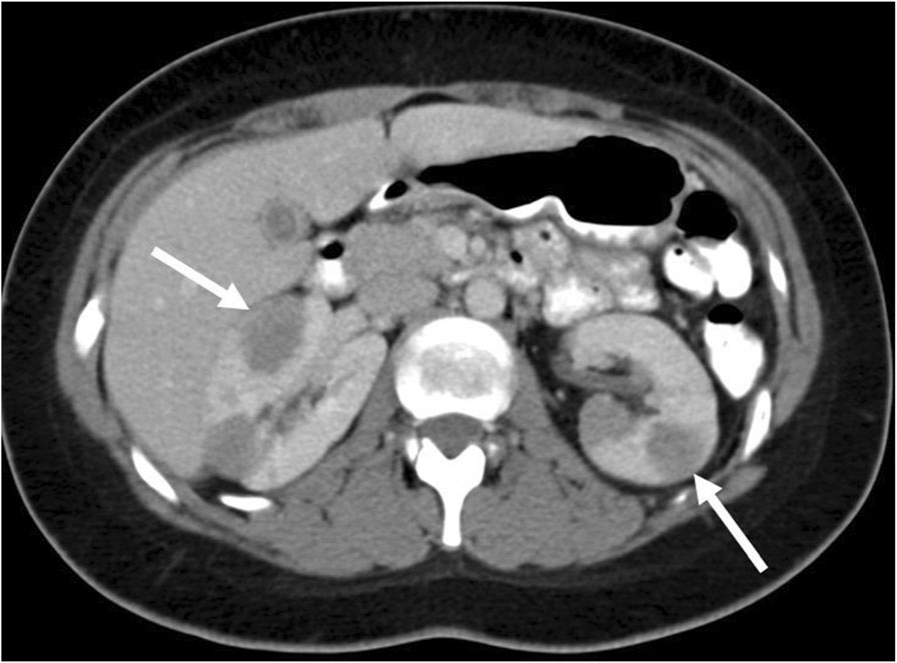
Renal involvement with renal masses. Contrast-enhanced axial CT image of a 23-year-old female with known Burkitt lymphoma demonstrates multiple circumscribed hypoattenuating renal masses (arrows)

**Fig. 6:**
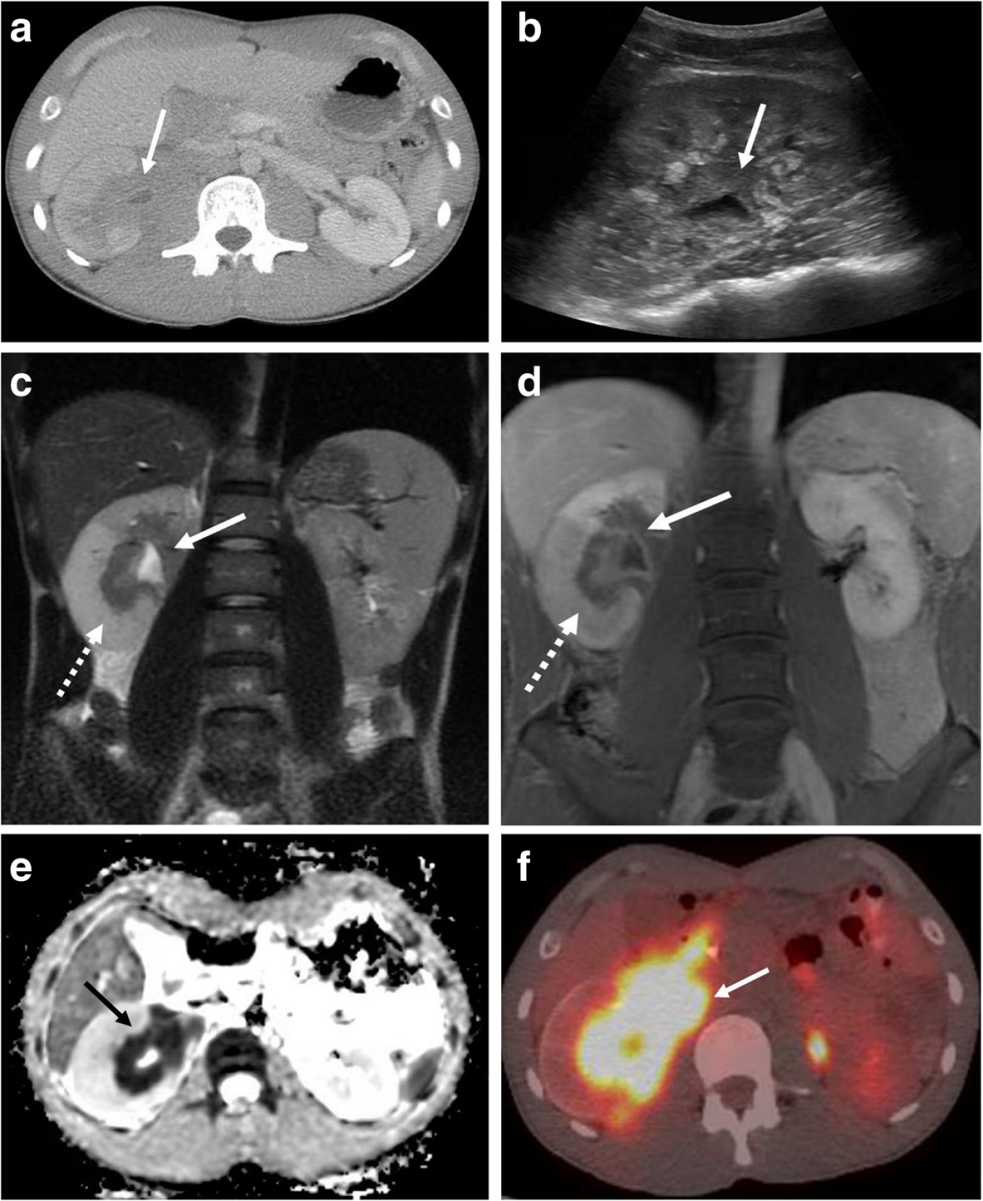
Renal collecting system involvement. A 24-year-old male presenting with abdominal and lower back pain. **a** Contrast-enhanced axial CT image demonstrates an infiltrative soft tissue mass (arrow) encasing the right renal collecting system with resultant dilated renal pelvis and urothelial thickening. **b** Corresponding sagittal grayscale ultrasound image (**b**) demonstrates obliteration of the normal renal sinus fat by hypoechoic tissue encasing the renal pelvis (arrow). Follow-up MRI with (**c**) coronal T2-weighted, (**d**) coronal T1-weighted post contrast, and (**e**) axial apparent diffusion coefficient images shows a right renal pelvis mass with associated diffusion restriction (solid arrows) with edema and delayed enhancement of portions of the right renal parenchyma (dashed arrows). **f** Follow-up fused axial PET-CT demonstrates associated hypermetabolic activity (arrow)

**Fig. 7:**
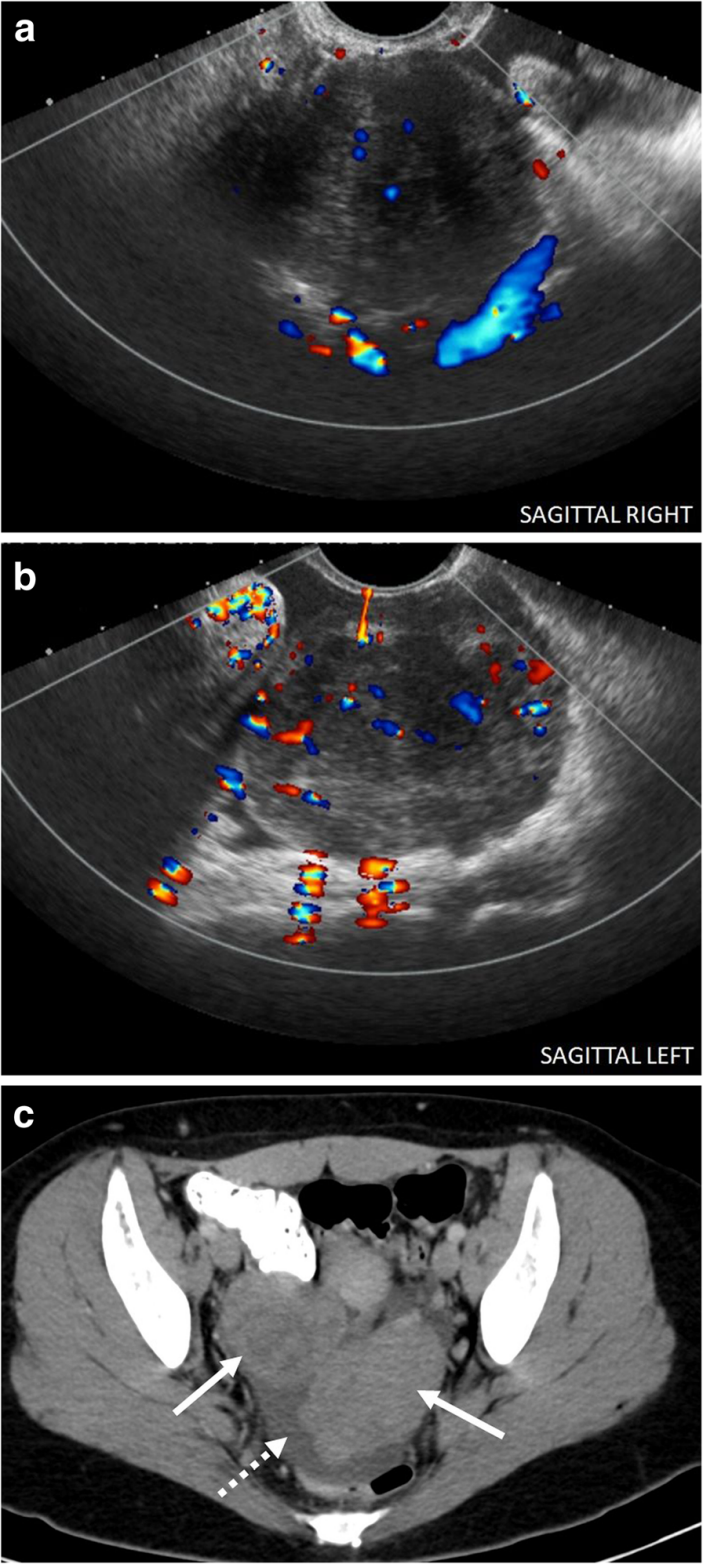
Bilateral ovarian masses. A 23-year-old female presenting with pelvic pain. Sagittal color Doppler images through the (**a**) right and (**b**) left ovaries demonstrate heterogeneous enlargement of the bilateral ovaries containing internal flow, greater on the left. **c** Subsequent contrast-enhanced CT shows markedly enlarged bilateral ovaries (solid arrows) with adjacent pelvic free fluid (dashed arrow). Subsequent exploratory laparotomy with bilateral salpingo-oophorectomy revealed Burkitt lymphoma

**Fig. 8:**
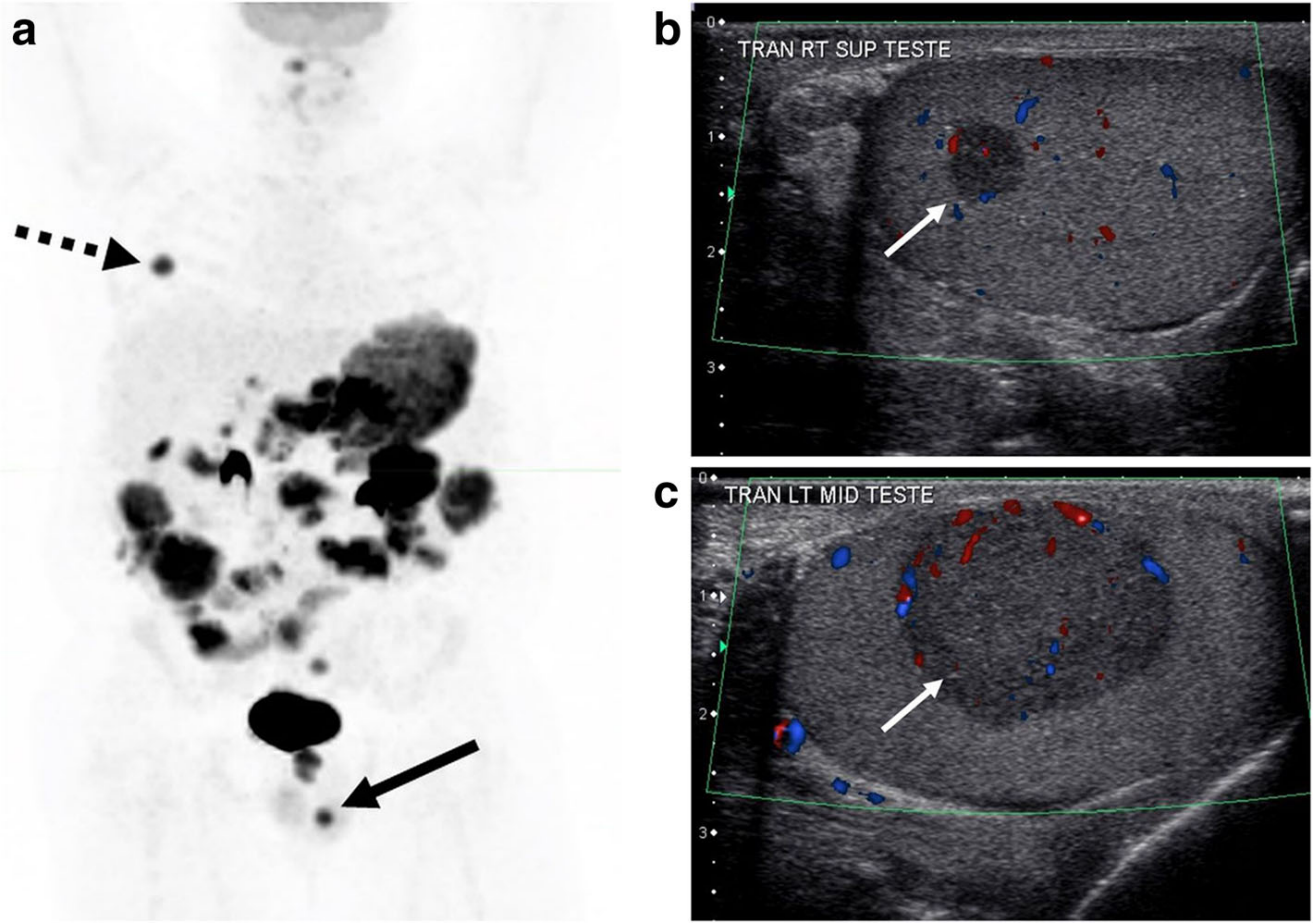
Bilateral testicular masses. A 39-year-old HIV-positive male with known Burkitt lymphoma. **a** Whole body planar PET image demonstrates widespread abdominal disease with a small focus of hypermetabolic activity within the region of the left testicle (solid arrow). Less intense, linear activity within the abdomen corresponds to physiologic bowel activity. Small focus of uptake in the right hemithorax (dashed arrow) corresponds to pleural disease. Sagittal color Doppler images through the (**b**) right and (**c**) left testicles demonstrate bilateral hypoechoic testicular masses, left larger than right (arrows)

Although less common than bowel involvement, solid organ involvement in the abdomen and pelvis may also occur [28]. BL of the liver is more often secondary than primary and may appear as single or multiple mass-like or infiltrative areas within the liver. Spleen involvement most commonly manifests as splenomegaly, although may be normal in size. Ultrasound is a useful means of monitoring spleen size, and PET/CT is highly accurate in detecting splenic involvement [31]. BL of the pancreas is uncommon but may present as biliary obstruction in cases involving the pancreatic head. Renal involvement is variable ranging from nephromegaly with striated nephrogram on CT or MRI with diffusely infiltrative disease (most common) or single or multiple discrete renal masses (Fig. 5). BL of the renal collecting system and ureter may present with hydronephrosis and renal failure (Fig. 6). Gonadal involvement is uncommon. In males, BL of the testicles may present as diffuse testicular enlargement or discrete testicular masses (Fig. 7). The epididymis and spermatic cord may also be involved. Appearance of ovarian BL is variable with reports of unilateral or bilateral cystic, solid, or mixed solid and cystic masses (Fig. 8) [12].

### Chest

**Fig. 9:**
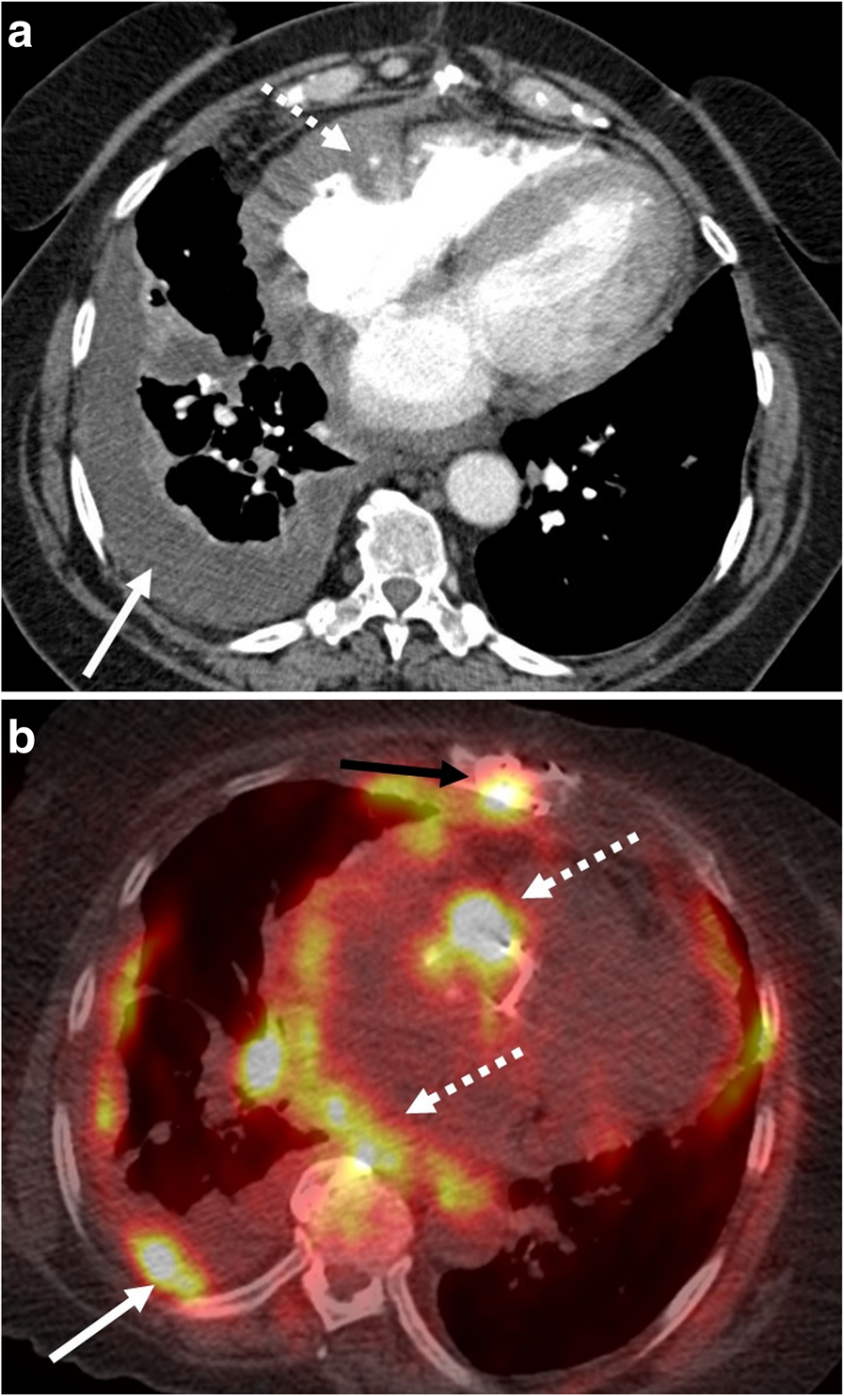
Pleural and pericardial involvement. A 62-year-old female presenting with chest pain and shortness of breath. **a** Contrast-enhanced axial CT image demonstrates a partially loculated right pleural effusion (solid arrow) and mass-like thickening of the pericardium extending into the epicardial fat encasing the right coronary artery (dashed arrow). **b** Fused axial PET-CT demonstrates associated hypermetabolic uptake within the pericardial mass (white dashed arrows) and better depicts multifocal right-sided pleural involvement (white solid arrow). There is also sternal involvement (black solid arrow)

**Fig. 10:**
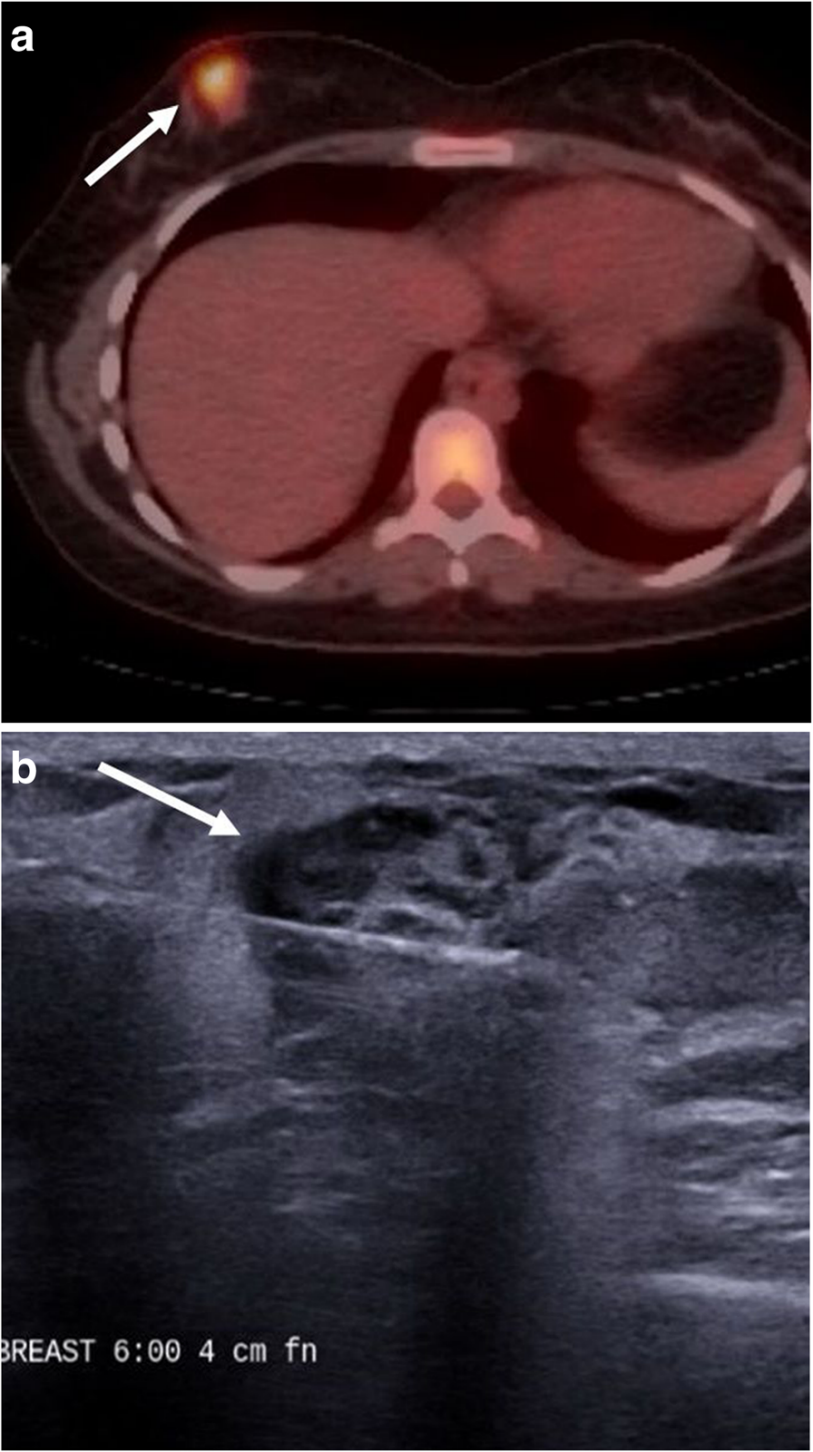
Breast involvement. A 22-year-old female with known Burkitt lymphoma. **a** Staging fused axial PET-CT demonstrates hypermetabolic activity within the inferior and medial right breast (arrow). **b** Grayscale ultrasound during percutaneous biopsy demonstrates an irregular, heterogeneous mass with indistinct margins (arrow). Biopsy results revealed Burkitt lymphoma

Thoracic involvement is common and most often manifests with pleural effusion, which can sometimes be massive, causing mediastinal shift [28]. Mediastinal or hilar lymphadenopathy are also seen [28, 32]. Uncommon intrathoracic manifestations of BL include pericardial and myocardial involvement, presenting with single or multiple masses, lung parenchymal and endobronchial lesions, and chest wall masses [33–35] (Fig. 9). Pleural disease usually presents as single or multiple pleural-based nodules and masses and may manifest symptomatically with dyspnea in the setting of a pleural effusion. Emergent presentations with cardiac tamponade appearing as a large pericardial effusion with cardiac chamber distortion and signs of reduced cardiac output have been reported with pericardial involvement [36]. BL of the breast is rare and is most commonly described as a rapidly growing, unilateral circumscribed breast mass, although bilateral and diffuse involvement has been reported [37] (Fig. 10).

### Head and neck and central nervous system

Head and neck involvement of BL is the characteristic and most common site of involvement in the endemic form of BL, occurring in up to 50–60% of cases and presenting clinically as a rapidly enlarging, often painless, jaw mass [38]. In the endemic form, the mandible and other facial bones are involved by osteolytic lesions with bony destruction and possible invasion of adjacent facial compartments such as the orbit. Adjacent nerves may be compromised presenting clinically with facial anesthesia or paresthesia.

**Fig. 11:**
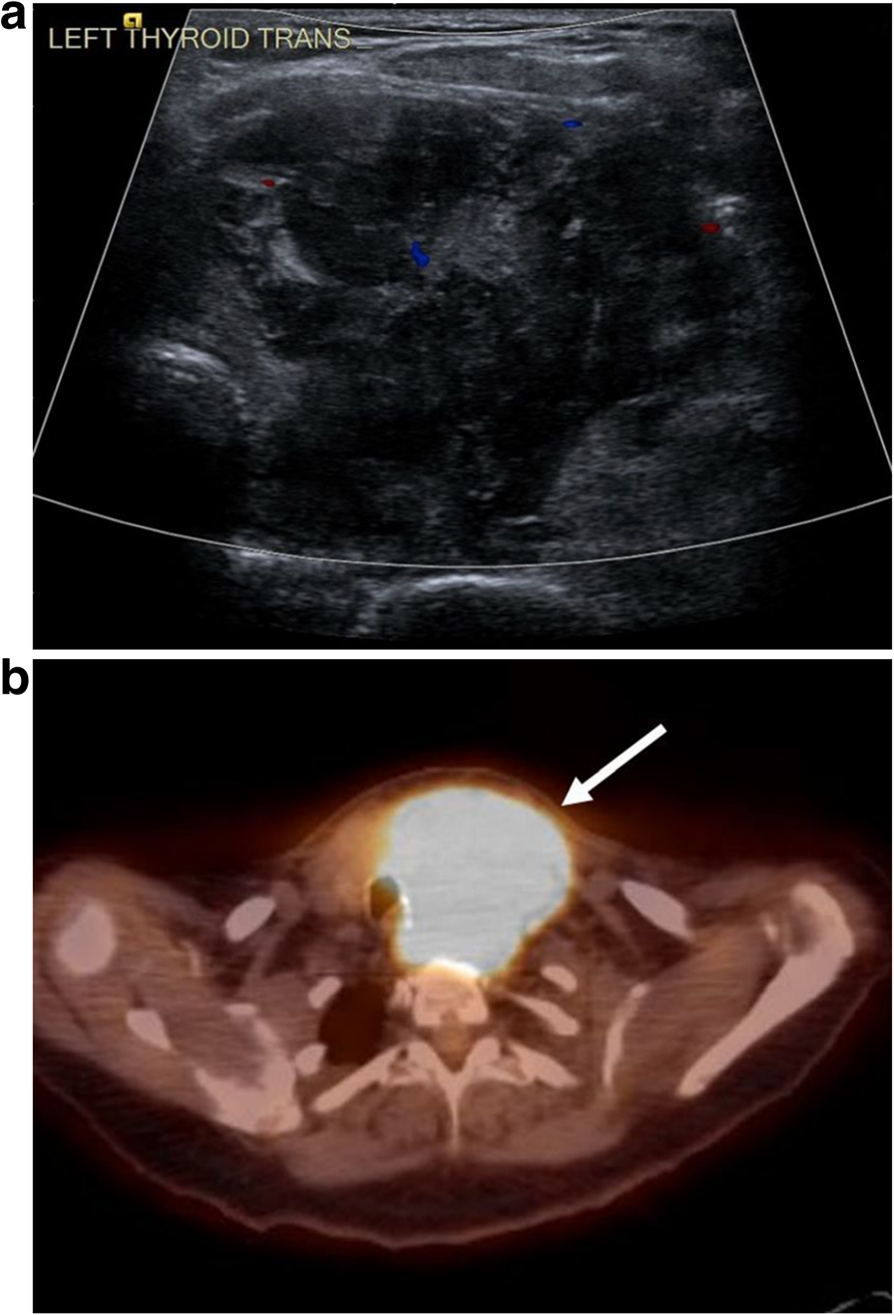
Thyroid involvement. A 58-year-old female presenting with dysphagia. **a** Transverse color Doppler image through the left thyroid demonstrates a large, heterogeneous hypovascular mass. **b** Fused axial PET-CT demonstrates the thyroid mass with rightward tracheal displacement and associated hypermetabolic activity. Biopsy of the thyroid mass revealed Burkitt lymphoma

Nodal involvement, on the other hand, is more often seen in sporadic BL and appears as mass-like, often heterogeneous, enlargement of the affected nodes. Extranodal structures of the head and neck most likely to be involved in sporadic BL are those of Waldeyer ring, which include the palatine and lingual tonsils and adenoids of the nasopharynx [39]. BL of the thyroid is rare but may manifest as thyroid nodules sometimes large enough to cause tracheal or neurovascular compression (Fig. 11) [40]. In contrast to endemic BL, nodal and extranodal masses in sporadic BL infiltrate, rather than erode, into adjacent facial compartments. While superficial nodal involvement may be depicted on ultrasound, the full extent of involvement and relationship to surrounding structures is better depicted on MRI and appears relatively homogenous with intermediate to low signal intensity on T2-weighted images and associated diffusion restriction.

**Fig. 12:**
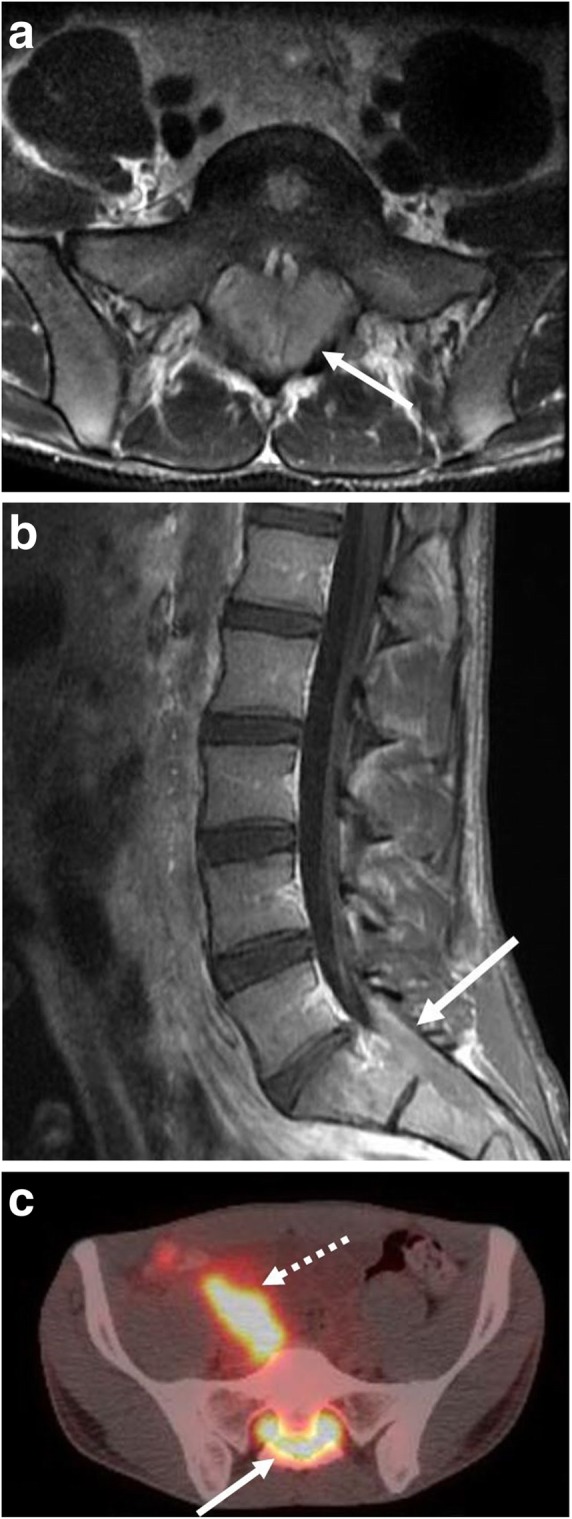
Central nervous system involvement. A 24-year-old male presenting with abdominal and lower back pain. **a** Axial T2-weighted and (**b**) sagittal post contrast images of the lumbar spine and sacrum demonstrate an enhancing soft tissue mass within the lower spinal canal (arrows) with extension into bilateral sacral neural foramina. **c** Fused axial PET-CT demonstrates associated hypermetabolic activity within the sacral mass (solid arrow). Involvement of the right retroperitoneum is also seen (dashed arrow)

Central nervous system involvement is also more common in sporadic and immunodeficiency-associated BL. BL of the CNS is seen in up to 15% of cases at presentation, is a common complication of recurrent or treatment resistant disease, and is associated with a worse prognosis [41]. CNS BL presents anywhere along the neuroaxis including within the brain and spinal canal and may present as a discrete mass or areas of leptomeningeal thickening (Fig. 12). However, even if CNS involvement is not apparent on imaging, lumbar puncture is necessary to exclude CNS BL.

### Bone marrow

**Fig. 13:**
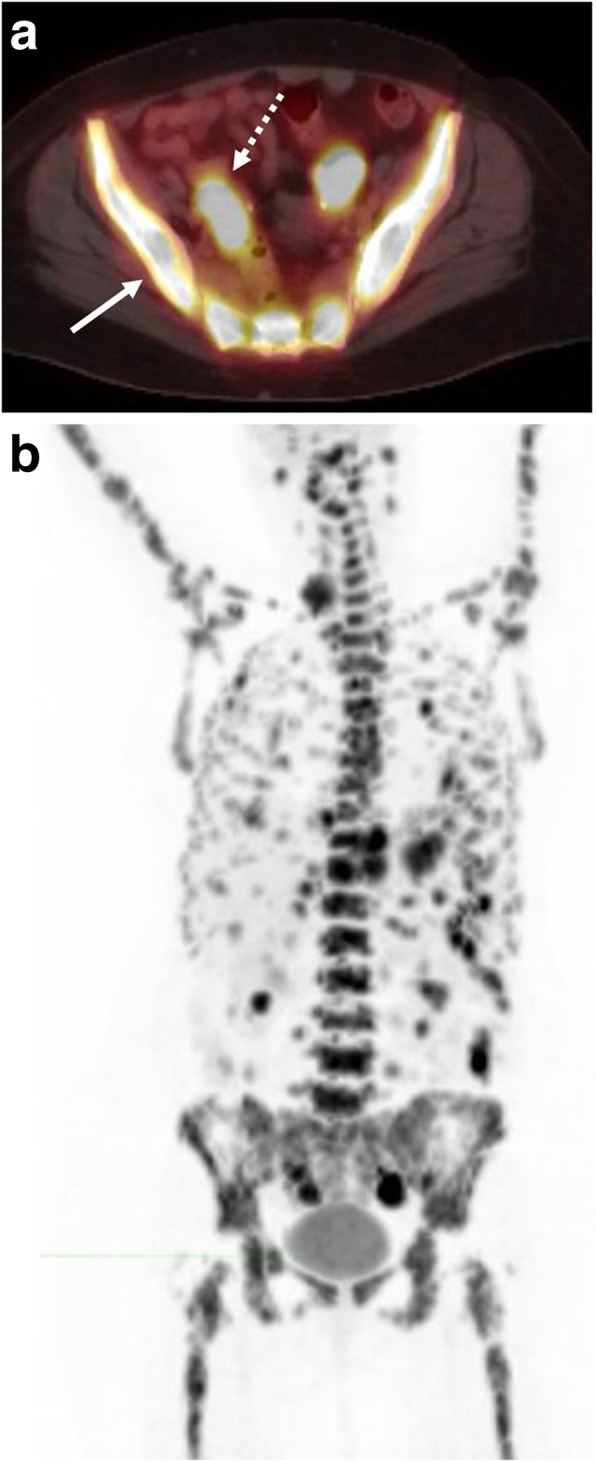
Diffuse marrow involvement. A 74-year-old female with known Burkitt lymphoma. **a** Fused axial PET-CT demonstrates diffuse hypermetabolic activity within the visualized marrow of the pelvis (solid arrow). Soft tissue activity is also seen within the pelvis (dashed arrow). **b** Whole body planar PET image demonstrates both diffuse marrow and soft tissue disease within the lower neck, thorax, abdomen, and pelvis

BL of the bone marrow is seen in approximately 30% of cases at presentation and, similar to CNS involvement, is seen in recurrent or treatment resistant disease, confers an advanced stage of disease, and associated with worse prognosis (Fig. 13) [41, 42]. On radiography and CT, lymphoma appears as a permeative lytic lesion with associated periosteal reaction and cortical destruction. On MRI, bone marrow involvement manifests as focal or diffuse areas of marrow replacement by low signal on T1-weighted images with associated increased T2 signal intensity. MRI often is better able to depict an associated soft tissue mass, if present. While BL of the bone marrow is often seen in the setting of bulky disease elsewhere, a subset of patients presents with bone marrow- and blood-predominant or exclusive disease, which is termed Burkitt leukemia and considered a variant of Burkitt lymphoma in the 2016 WHO classification [10]. Burkitt leukemia patients with isolated bone marrow involvement have a better prognosis compared to BL patients with bone marrow involvement when treated similarly [43]. Analogous to BL of the CNS, bone marrow biopsy is necessary to exclude bone marrow involvement not visible on imaging.

## Treatment

**Fig. 14:**
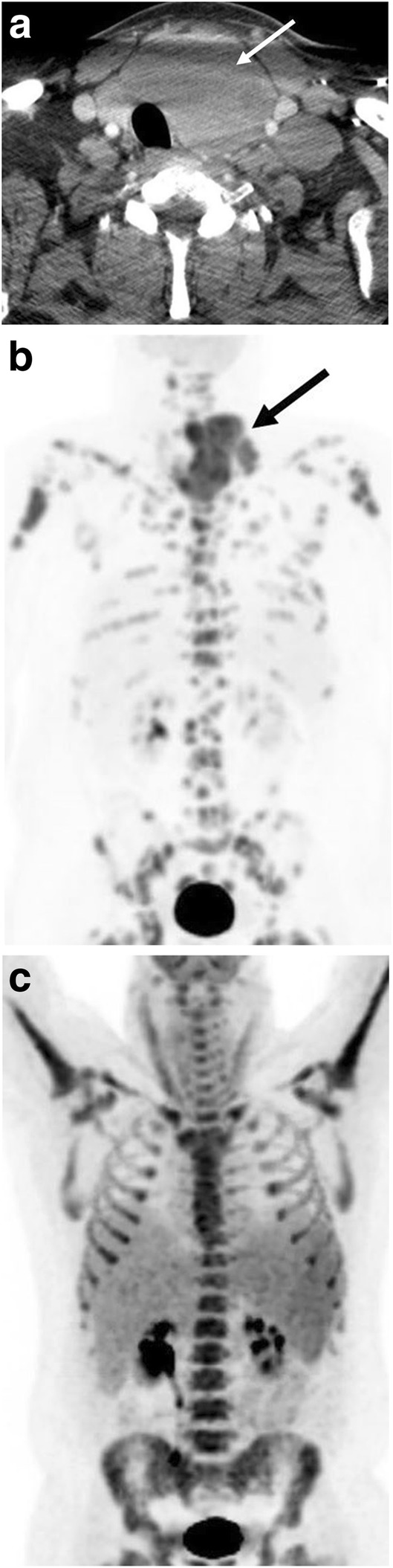
Treatment response. A 42-year-old man initially presenting with neck mass and constitutional symptoms. Contrast-enhanced axial CT at presentation (**a**) demonstrates a large left neck mass (arrow) replacing the left thyroid with tracheal deviation. **b** Staging whole body planar PET image demonstrates a hypermetabolic left lower neck mass (arrow) and heterogeneous marrow involvement. The patient underwent R-CHOP with intrathecal methotrexate, 4 cycles of hyper-CVAD and intrathecal methotrexate. Follow-up (**c**) whole-body planar PET image imaging show resolution of the cervical mass without evidence of distant disease. Diffuse homogeneous marrow uptake on the PET scan is likely related to post-therapy marrow activation

Chemotherapy is the mainstay of therapy in BL (Fig. 14). Given the effectiveness of chemotherapy regimens and often widespread disease at presentation, there is no role for radiation therapy. Similarly, even in localized cases, surgery is generally not pursued unless disease complications necessitate prompt surgical intervention such as in cases of bowel obstruction [44]. The approach to therapy in the different subtypes of BL is similar.

### Treatment protocols

Given the lack of randomized control trials, there is currently no clear choice of therapy in BL. Furthermore, most adult BL regimens are based on pediatric clinical trials. Current treatment regimens, either short or longer duration, employ intensive, multi-agent regimens composed of doxorubicin, alkylators, vincristine, and etoposide [45]. Less intensive regimens (i.e., CHOP-based therapies) used in other subtypes of non-Hodgkin lymphoma have been associated with higher rates of disease relapse [46]. Many current regimens incorporate the use of rituximab, often starting in the second cycle to minimize adverse therapy effects. In a multi-center trial of 260 patients with median follow-up of 38 months, Ribrag et al. demonstrated improved event free (75% vs 62%) and overall (83% vs 70%) survival at 3 years without significant difference in adverse events [47]. CNS prophylaxis with intrathecal methotrexate and/or cytarabine is also a component of first-line therapy given high risk of CNS involvement in BL patients. Without CNS prophylaxis, between 30 and 50% will develop CNS relapse, often within the first year, with rates decreasing to 6–11% with CNS prophylaxis [48–50].

### Surveillance and refractory disease

Following initiation of therapy, patients are followed regularly and assessed via history, physical examination, and laboratory studies including complete blood count, lactate dehydrogenase, and biochemical profile. In adults, imaging surveillance with CT is performed, as described previously. In children, routine surveillance with cross-sectional imaging may not be warranted for patient achieving complete response after therapy given overall low rates of relapse along with increased costs and added risks of radiation exposure [51]. Thus, surveillance may be performed with cervical and abdominal ultrasound with chest radiography with CT or PET/CT reserved if there are signs of relapse. Given recent advances in therapy, the overall survival in BL has continued to improve. Patients less than 19 years old have the best prognosis with 87% 5-year survival for diagnoses between 2002 and 2008, while older patients and those advanced disease have the worst prognosis. For example, BL patients diagnosed after the age of 60 years have a 5-year survival rate of 33% [52].

**Fig. 15:**
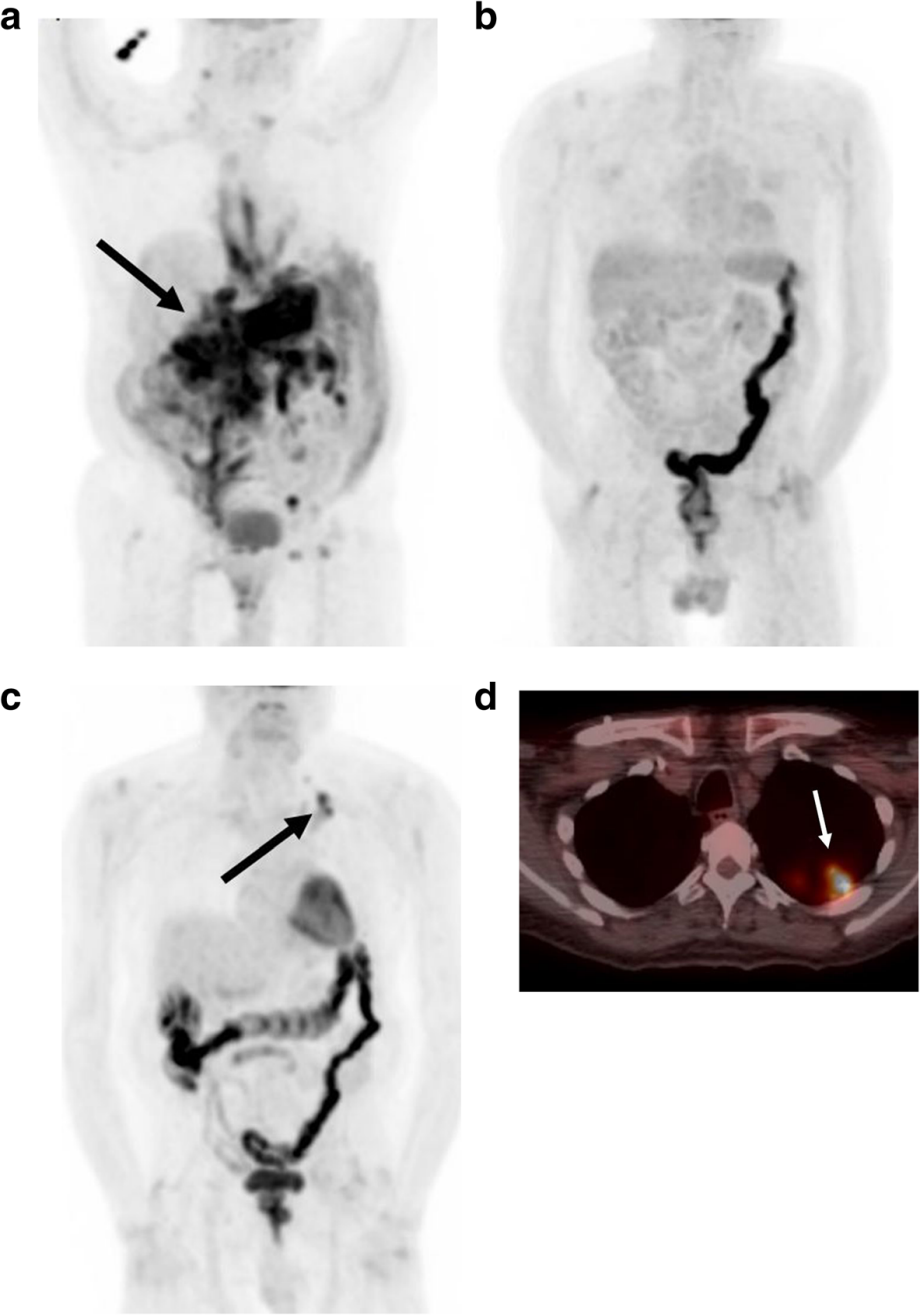
Pulmonary recurrence. An 83-year-old man initially presenting with GI bleeding and was found to have conglomerate gastric and intra-abdominal masses. **a** Initial staging whole body planar PET image demonstrates extensive abdominal disease involvement (arrow). He went on to complete 4 cycles of bendamustine and rituximab. **b** Follow-up whole body planar PET image demonstrates complete disease response. **c** A subsequent restaging whole body planar PET image demonstrates a new hypermetabolic focus within the left upper thorax (arrow). **d** Corresponding fused axial PET-CT demonstrates a pleural-based lesion (arrow). Percutaneous biopsy revealed Burkitt lymphoma

Patients with relapsed disease usually present symptomatically and within the first year after the completion of treatment. Relapsed disease may occur at the sites of original presentation or remotely (Fig. 15). Currently, no prospective studies have evaluated therapy regimens in relapsed patients, and patients who have failed first-line therapy often undergo salvage chemotherapy as a part of clinical trials [45]. Relapsed patients may also undergo autologous stem cell transplantation [53].

### Treatment-related complications

**Fig. 16:**
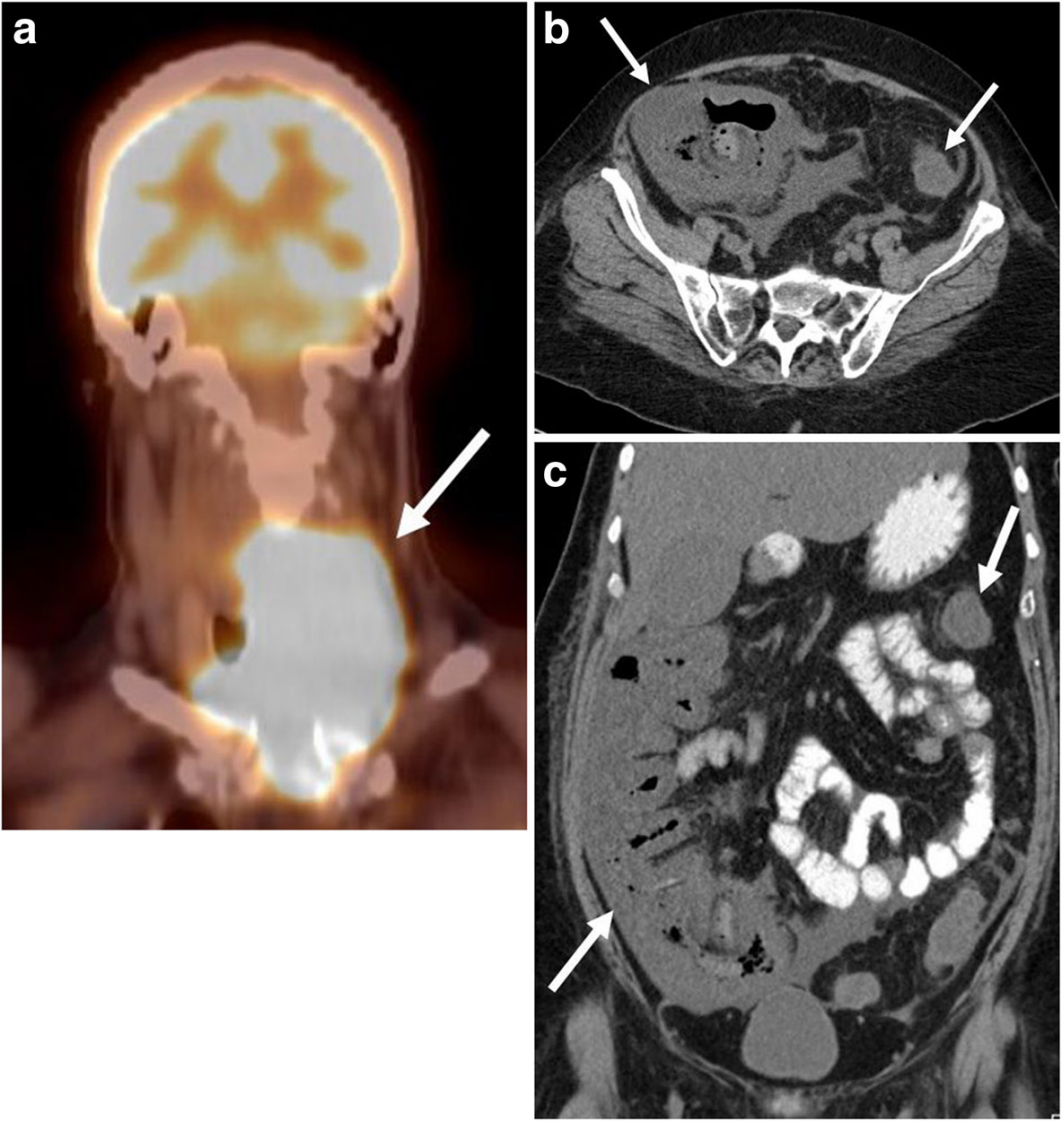
Treatment-related neutropenic colitis. A 58-year-old male initially presenting with a left neck mass. **a** Coronal fused PET-CT demonstrates a large, hypermetabolic left neck mass (arrow) causing tracheal deviation. The mass was subsequently biopsied as Burkitt lymphoma, and the patient was subsequently started on hyper-CVAD therapy. After cycle #3, the patient was admitted for abdominal pain and neutropenic fever. **b** Axial and (**c**) coronal non-contrast CT images obtained in the emergency department show diffuse colonic wall thickening and inflammatory stranding (arrows). Findings are consistent with treatment-related colitis

Given the high dose, intensive chemotherapy regimens employed during BL, treatment-related toxicities and complications are commonly encountered clinically. Regardless of treatment regimen, tumor lysis syndrome (TLS) is a potential complication of therapy due to rapid growth rates of tumor cells. TLS is caused by release of cellular products overwhelming the kidneys’ excretory capacity, leading to electrolyte imbalances including hyperkalemia, hyperphosphatemia, hyperuricemia, and ultimately renal failure. Prophylaxis and treatment of TLS include intravenous hydration, use of hypouricemic agents such as allopurinol and rasburicase, and, when indicated, dialysis. Furthermore, complications specific to various chemotherapeutic agents and regimens may also arise during treatment, many of which may be detected on imaging. For example, methotrexate is associated with pneumonitis presenting as intestinal and airspace infiltrates. Neutropenic colitis may manifest with any agent causing severe neutropenia, which presents with colonic wall thickening and edema typically affecting the cecum and ascending colon (Fig. 16). Cyclophosphamide is associated with the development of hemorrhagic cystitis, which appears as diffuse thickening and nodularity of the bladder wall [54].

## Conclusion

BL is a highly aggressive, rapidly growing B cell non-Hodgkin lymphoma. BL has a very heterogeneous pattern of presentation and clinical course. Given the diverse nature of disease involvement, multiple imaging modalities play a key role in Burkitt lymphoma evaluation throughout the entire disease course, each with certain advantages and disadvantages. Furthermore, radiologists should recognize common and atypical presentations and sites of disease to appropriately guide clinicians given the urgency of potential treatment.

## Abbreviations

BL: Burkitt lymphoma
CNS: Central nervous system
CT: Computed tomography
DLBCL: Diffuse large B cell lymphoma
DWI: Diffusion-weighted imaging
EBV: Epstein-Barr virus
MUGA: Multigated
NCCN: National Comprehensive Cancer Network
NHL: Non-Hodgkin lymphoma
PET: Positron emission tomography
SUV: Standardized uptake value
TLS: Tumor lysis syndrome
WHO: World Health Organization
